# Modest effect on plaque progression and vasodilatory function in atherosclerosis-prone mice exposed to nanosized TiO_2_

**DOI:** 10.1186/1743-8977-8-32

**Published:** 2011-11-10

**Authors:** Lone Mikkelsen, Majid Sheykhzade, Keld A Jensen, Anne T Saber, Nicklas R Jacobsen, Ulla Vogel, Håkan Wallin, Steffen Loft, Peter Møller

**Affiliations:** 1 Department of Public Health, University of Copenhagen, 1014 Copenhagen K, Denmark; 2 Department of Pharmacology and Pharmacotherapy, University of Copenhagen, 2100 Copenhagen Ø, Denmark; 3 National Research Centre for the Working Environment, 2100 Copenhagen Ø, Denmark

## Abstract

**Background:**

There is growing evidence that exposure to small size particulate matter increases the risk of developing cardiovascular disease.

**Methods:**

We investigated plaque progression and vasodilatory function in apolipoprotein E knockout (*ApoE*^-/-^) mice exposed to TiO_2_. *ApoE*^-/- ^mice were intratracheally instilled (0.5 mg/kg bodyweight) with rutile fine TiO_2 _(fTiO_2_, 288 nm), photocatalytic 92/8 anatase/rutile TiO_2 _(pTiO_2_, 12 nm), or rutile nano TiO_2 _(nTiO_2_, 21.6 nm) at 26 and 2 hours before measurement of vasodilatory function in aorta segments mounted in myographs. The progression of atherosclerotic plaques in aorta was assessed in mice exposed to nanosized TiO_2 _(0.5 mg/kg bodyweight) once a week for 4 weeks. We measured mRNA levels of *Mcp-1*, *Mip-2*, *Vcam-1*, *Icam-1 *and *Vegf *in lung tissue to assess pulmonary inflammation and vascular function. TiO_2_-induced alterations in nitric oxide (NO) production were assessed in human umbilical vein endothelial cells (HUVECs).

**Results:**

The exposure to nTiO_2 _was associated with a modest increase in plaque progression in aorta, whereas there were unaltered vasodilatory function and expression levels of *Mcp-1*, *Mip-2*, *Vcam-1*, *Icam-1 *and *Vegf *in lung tissue. The *ApoE^-/- ^*mice exposed to fine and photocatalytic TiO_2 _had unaltered vasodilatory function and lung tissue inflammatory gene expression. The unaltered NO-dependent vasodilatory function was supported by observations in HUVECs where the NO production was only increased by exposure to nTiO_2_.

**Conclusion:**

Repeated exposure to nanosized TiO_2 _particles was associated with modest plaque progression in *ApoE^-/- ^*mice. There were no associations between the pulmonary TiO_2 _exposure and inflammation or vasodilatory dysfunction.

## Introduction

The investigation of toxicological effects of nanoparticles is increasingly important due to their growing occupational use and presence as compounds in consumer products. The use of nanometer-size particles in paints can reduce the production costs by the addition of lower (mass) concentrations or add novel properties to the final product. TiO_2 _pigments are widely used by paint and plastic industries for whiteness and opacity. One of the major advantages of TiO_2 _is its resistance to discoloration by UV light. TiO_2 _particles are considered to have low toxicity to humans and animals [1]. However, it has been shown that decreasing particle size is associated with increased pro-inflammatory properties [2]. An important possible health risk of particle exposure is cardiovascular effects. It has been shown that exposure to combustion-based particles in ambient air is associated with progression of atherosclerosis, myocardial infarction and cardiovascular mortality in humans and the nanosized fraction is considered an important culprit [3-5]. The mechanisms of these effects are considered to involve oxidative stress and inflammation, vasomotor dysfunction, neuronal signalling and possible translocation of particles from the airways to the circulation [3]. Pulmonary exposure to carbon-based particles can accelerate plaques progression in animal models [6,7]. However, associations between exposure to TiO_2 _particles and progression of atherosclerosis have not been assessed, whereas pulmonary exposure has been associated with vasomotor dysfunction [8].

Endothelial cells have an important barrier function and produce factors that regulate vascular tone, cellular adhesion, smooth muscle cell proliferation, vessel wall inflammation, and thromboresistance [9,10]. In the functional endothelium, acetylcholine binds to muscarinic receptors on the luminal surface leading to nitric oxide (NO) production by the calcium dependent, constitutive isoform of NO synthase (eNOS) [9]. Impaired endothelium-dependent vasodilation is a central feature of endothelial dysfunction and is associated with increased risk of developing cardiovascular diseases [11]. Endothelial dysfunction is associated with increased levels of proinflammatory factors, such as adhesion molecules and chemoattractants [12], which can lead to atherosclerosis [9,11,13]. This is attributed to increased intravascular generation of reactive oxygen species (ROS) such as superoxide anion radicals, leading to reduced bioavailability of NO [12]. Superoxide dismutase (SOD) converts superoxide anion radicals to hydrogen peroxide, which may ameliorate the production of peroxynitrite [12]. *Ex vivo *exposure of isolated vessels to the exogenous SOD mimic, tempol, has been associated with improved endothelium-dependent vasodilation in arteries from hypertensive rats, most likely by reduced intracellular ROS production by tempol that diffuses freely across cell membranes [12,14].

Inflammation plays a key role in atherogenesis as well as destabilization of plaques [15]. According to the multistep theory, monocytes adhere to the endothelial cells by binding to intracellular adhesion molecule-1 (ICAM-1) and vascular cell adhesion molecule-1 (VCAM-1), and then migrate into the intima [16,17]. Monocyte chemoattractant protein-1 (MCP-1/CCL2) is a chemotactic cytokine produced by endothelial cells after exposure to cytokines and oxidized lipoproteins. MCP-1 plays an important role in the migration and activation of monocytes and T cells and regulates the proliferation of vascular smooth muscle cells [18]. Activated macrophages express proinflammatory cytokines including macrophage inflammatory protein 2 (MIP-2/CXCL2) [19]. This cytokine up-regulates the expression of ICAM-1 and VCAM-1 on the endothelial cell surface. Vascular endothelial growth factor (VEGF) stimulates the proliferation and growth of endothelial cells and additionally increases vascular permeability [20].

We hypothesised that pulmonary exposure to nanosized particles, compared to fine particle would evoke larger cardiovascular effects, whereas altered vasodilatory function would be blunted by *ex vivo *treatment with the superoxide dismutase mimic tempol. To this end we investigate plaque progression and vasodilatory function in dyslipidemic and atherosclerosis prone apolipoprotein E knockout (*ApoE*^-/-^) mice exposed by intratracheal instillation (i.t.) to three physicochemically different TiO_2 _particles including nanometer-size coated rutile TiO_2 _(nTiO_2_; 20.6 nm), nanometer-size and highly photocatalytic anatase-rich TiO_2 _(pTiO_2_; 12 nm), and sub-micrometer-size coated rutile TiO_2 _(fTiO_2_; 288 nm). Inflammation was evaluated by measuring mRNA levels of *Mip2*, *Icam1*, *Vcam1*, *Mcp1*, and *Vegf *in lung tissue. We also studied the NO production in human umbilical vein endothelial cells (HUVECs) exposed to the three TiO_2 _particles.

## Results

### Particle and exposure characterization

The particles had different crystalline form, crystallite size, surface area, coating and chemistry (table 1). The fTiO_2 _and nTiO_2 _were mainly rutile crystal structure. The fTiO_2 _particles were coated with Al_2_O_3 _(3.22 wt%) and polyol (1.3 wt%). The nTiO_2 _particles were coated with SiO_2 _(12.01 wt%), Al_2_O_3 _SiO_2 _(4.58 wt%), ZrO_2 _(1.17 wt%) and polylol (5.2 wt%) [21,22]. The pTiO_2 _had anatase crystal structure; it was uncoated, highly pure and delivered as a suspension in water (30 wt%).

**Table 1:** Primary physicochemical characteristics of the particulate TiO_2 _materials and hydrodynamic sizes in exposure dispersions.

Electron Microscopy images	Sample material	Product name	Phase(s)	Crystallite size for primary particles (nm)	Surface area BET (m^2^/g)	Minor elements/surface coatings on the primary particles (wt%)	Particle size in exposure dispersions (nm ± SD)	
							
							unfiltered	3.0 μm filter
	Fine TiO_2 _(fTiO_2_)	RDI-S	99.5% rutile 0.5% anatase	288	21	Al_2_O: 3.22 P_2_O_5_: 0.12 ZrO_2_: 0.07 ^§^Polyol: 1.3	3415.8 ± 228.3	560.9 ± 162.0

	Photocatalytic TiO_2 _(pTiO_2_)	VP Disp. W 2730 X	7.8% rutile 92.2% anatase	19 12	N.A.	Al_2_O_3_: 0.10	NA	2320.5 ± 272.6

	NanoTiO_2 _(nTiO_2_)	UV-Titan L181	100% rutile	20.6	107.7	Na_2_O: 0.60 SiO_2_: 12.01 Al_2_O_3_: 4.58 ZrO_2_: 1.17 ^§^Polyol: 5.2	5223.5 ± 831.6	518.2 ± 118.2

^§ ^Compound according to the manufacturer

### Assessment of particle distribution in the lung of mice

**Figure 1:**
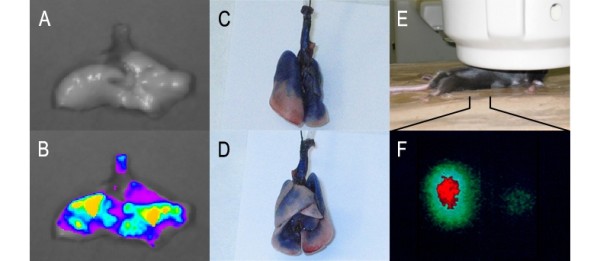
**Distribution of particles after i.t. instillation in wild-type mice**. Image A (front) and B (back) show the staining in the lung of mice after i.t. instillation of 1% Evans Blue solution (50 μl/mouse). Image C (no filter) and D (recorded as fluorescence passed through a 620 nm band pass filter) are images of the lung from a mouse after i.t. instillation of quantum dot QD621 solution (50 μl/mouse). Image E and F have been obtained in a single photon emission tomograph γ-camera in a mouse i.t. instilled with 18 nm nanogold particles.

We investigated the distribution of particles following i.t. instillation of Evans Blue, quantum dots and radioactively labelled gold nanoparticles in wild-type mice (Figure 1). The distribution of i.t. instilled particles was determined by three different test systems; this combination of the tests provides a reliable assessment of the distribution in whole lungs. The Evans blue staining served as real visual inspection of the instilled fluid, whereas the distribution of particles were visualised by quantum dots and radioactively labelled gold nanoparticles. Collectively, these results show that the i.t. instillation procedure yielded an even distribution in the lungs.

### Effect on vasodilatory response in aorta

Mice, aged 11-13 weeks, were exposed by i.t. instillation to either a control solution with 90% isotonic saline and 10% bronchoalveolar lavage (BAL) fluid, or particle (fTiO_2_, pTiO_2_, or nTiO_2_) suspended in 90% isotonic saline and 10% BAL fluid.

**Figure 2:**
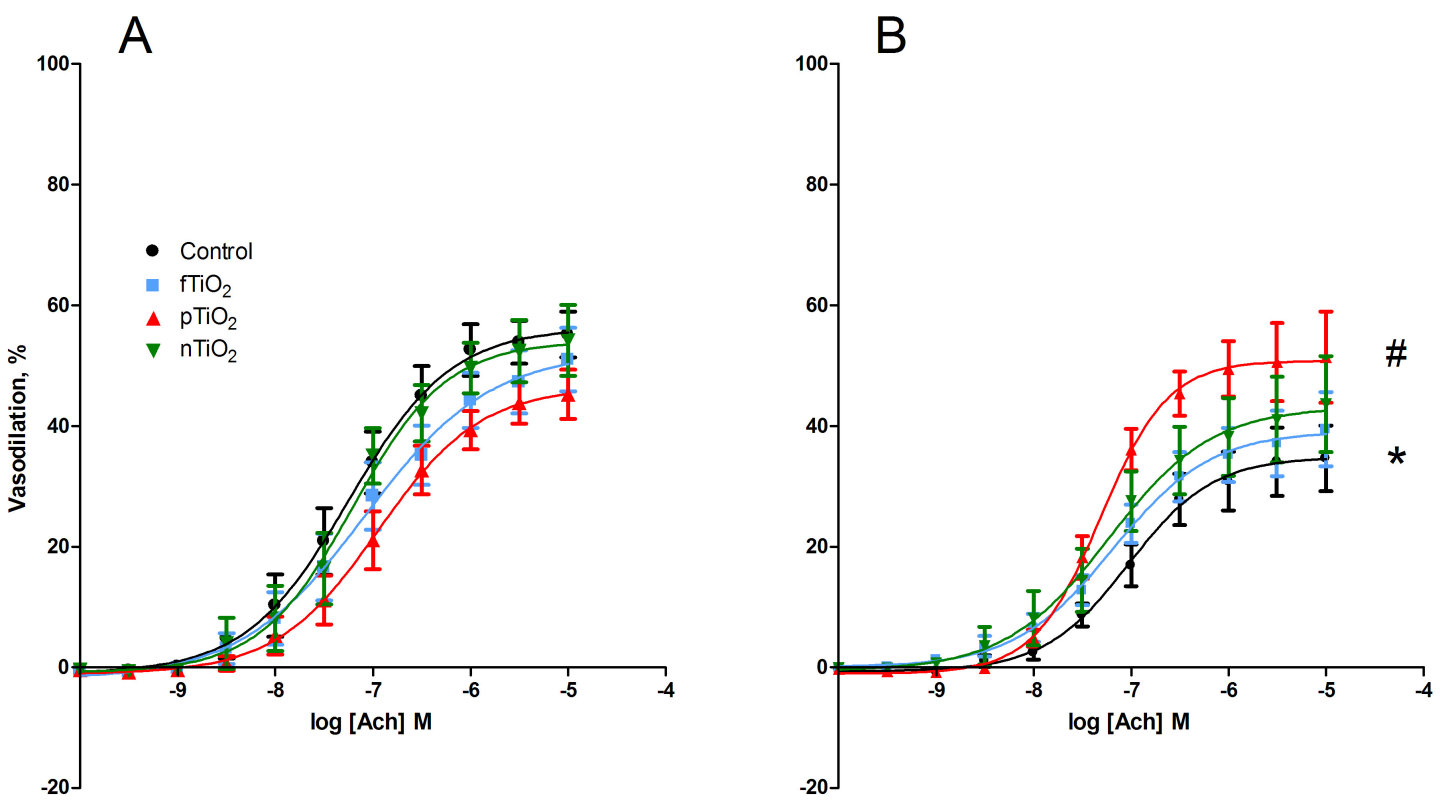
**Endothelium-dependent acetylcholine (ACh)-induced vasodilation of aorta segments from *ApoE^-/- ^*mice exposed by intratracheal instillation to control solution containing 90% saline and 10% BAL fluid (black), nTiO_2 _(green), fTiO_2 _(blue), or pTiO_2 _(red)**. The measurements are performed in A) absence or B) *ex vivo *presence of the SOD mimic, tempol. The response is expressed as the percent vasodilation of the pre-contraction tension produced by PGF_2_α. Each point on the curves represents the cumulative response at each concentration of the vasodilator. Data are expressed as means ± SEM (n = 10-11). * P < 0.05 (ANOVA) compared with E_max _in segments that were not treated with tempol. ^# ^P < 0.05 (ANOVA) compared with E_max _values in tempol-treated aorta segments of control mice.

The endothelium-dependent vasodilation induced by acetylcholine showed an interaction between the treatment with particles and tempol (P < 0.05, ANOVA). The post-hoc analysis of the interaction showed that the tempol treatment was associated with a 45% (95% CI: 19-71%) reduction of the E_max _value in animals i.t. instilled with control suspension. There were no differences in the E_max _values between the particle-exposed mice and controls for the aorta segments that were not treated with tempol. However, the E_max _value of tempol-treated vessels isolated from the animals exposed to pTiO_2 _particles was 71% (95% CI: 18 - 125%) higher than vessels isolated from mice exposed to the control suspension. There were no effects on EC_50 _values for the acetylcholine response (P = 0.83, single-factor effect of the particles) (table 2, Figure 2).

**Table 2:** Endothelium dependent (acetylcholine: ACh, and calcitonin-gene related peptide: CGRP) and independent (nitroglycerin: NTG, and felodipine: FD) vasodilatory function in *ApoE*^-/- ^mice after intratracheal instillation of TiO_2 _particles or control solution (90% saline and 10% BAL fluid).

Exposure i.t. instillation	Drug	- tempol		+ **tempol**	
		EC_50 _(nM)	E_max _(%)	EC_50 _(nM)	E_max _(%)
Control	**ACh**	160.4 ± 65.0	56.7 ± 3.59	105.9 ± 35.6	31.2 ± 5.39*
fTiO_2_		207.7 ± 75.9	51.8 ± 4.98	107.5 ± 48.9	39.9 ± 6.23
pTiO_2_		222.3 ± 67.6	46.1 ± 3.92	49.3 ± 7.73	53.5 ± 7.31^#^
nTiO_2_		101.7 ± 32.1	54.7 ± 4.99	64.2 ± 11.0	42.1 ± 7.43

Control	**CGRP**	8.7 ± 2.1	87.9 ± 2.69	13.3 ± 2.73	72.4 ± 5.82*
fTiO_2_		11.6 ± 2.1	82.7 ± 3.23	15.2 ± 2.28	73.6 ± 3.23
pTiO_2_		9.4 ± 0.90	90.0 ± 1.57	11.1 ± 0.53	78.2 ± 3.34
nTiO_2_		14.8 ± 2.1	80.4 ± 6.20	14.5 ± 2.42	70.9 ± 6.96

Control	**NTG**	23.8 ± 3.96	69.8 ± 3.41	68.7 ± 12.4*	60.3 ± 2.36*
fTiO_2_		25.8 ± 4.63	66.5 ± 2.87	64.1 ± 16.9	60.3 ± 3.37
pTiO_2_		25.30 ± 8.26	69.8 ± 2.97	41.7 ± 5.3	65.6 ± 4.30
nTiO_2_		21.3 ± 4.43	67.3 ± 3.03	55.1 ± 20.9	64.4 ± 2.09

Control	**FD**	10.4 ± 2.70	84.7 ± 3.74	7.57 ± 1.58	85.9 ± 2.69
fTiO_2_		6.67 ± 1.05	84.6 ± 4.62	5.96 ± 0.94	89.7 ± 2.36
pTiO_2_		6.55 ± 0.90	84.8 ± 1.87	5.26 ± 1.04	86.1 ± 1.81
nTiO_2_		7.82 ± 1.79	90.6 ± 2.70	8.58 ± 2.95	83.5 ± 2.83

All measurements were done in both absence and *ex vivo *presence of the SOD mimic, tempol. Data are expressed as means ± SEM (n = 10-11).

^# ^Interaction between the treatment with particles and tempol (P < 0.05, ANOVA). The post-hoc analysis showed lower E_max _values in animals intratracheally instilled with control solution, whereas there are higher E_max _values in *ex vivo *tempol treated vessels of the animals exposed to pTiO_2 _particles. *P < 0.05 (ANOVA) compared to E_max _values in segments that were not treated with tempol.

**Figure 3:**
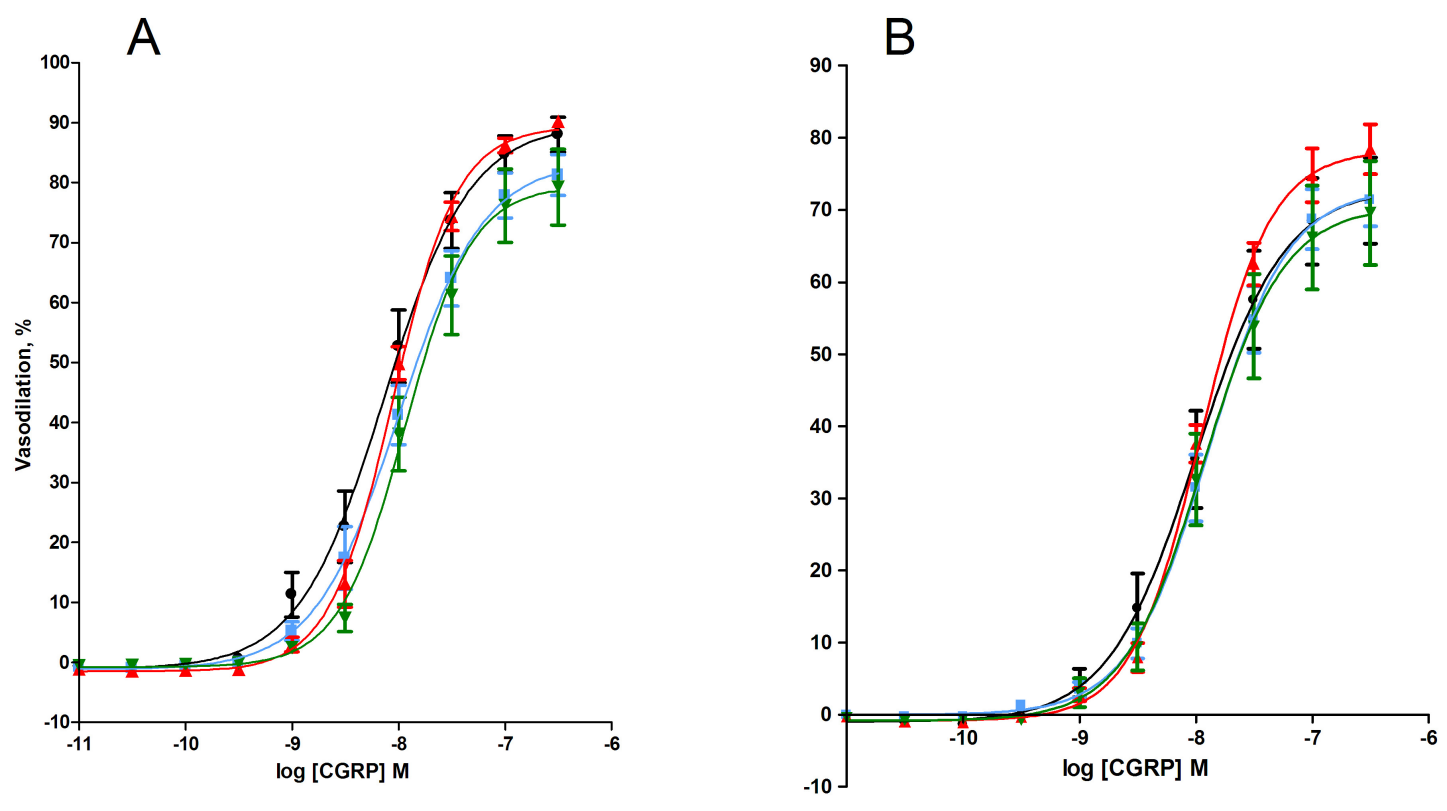
**Calcitonic-gene related peptide (CGRP)-induced vasodilation of aorta segments from *ApoE^-/- ^*mice exposed by intratracheal instillation to control solution consisting of 90% saline and 10% BAL fluid (black), nTiO_2 _(green), fTiO_2 _(blue), or pTiO_2 _(red)**. The measurements are performed in A) absence or B) *ex vivo *presence of the SOD mimic, tempol. The response is expressed as the percent vasodilation of the precontraction tension produced by PGF_2_α. Each point on the curves represents the cumulative response at each concentration of the vasodilator. Data are expressed as means ± SEM (n = 10-11). * P < 0.05 (ANOVA) compared with E_max _values in tempol-treated aorta segments of control mice.

The vasodilation was also assessed by stimulation with calcitonin-gene related peptide (CGRP) that activates CGRP receptors on aortic smooth muscle cells and endothelial cells [23]. There was no difference in the CGRP-mediated vasodilation between the particle-exposed mice and controls. The effect of CGRP showed an 18% (95% CI: 3.0 - 33%) reduction of the maximal response (E_max_) by *ex vivo *treatment with tempol, whereas there was no difference in terms of EC_50 _values (table 2, Figure 3).

**Figure 4:**
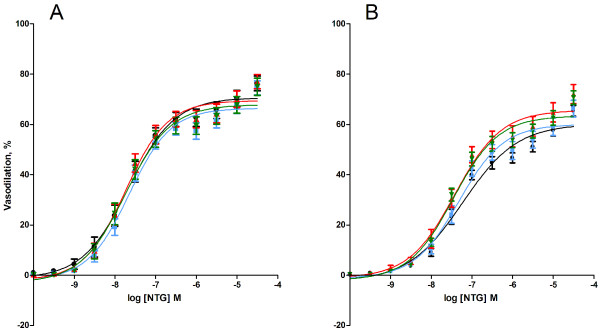
**Endothelium-independent nitroglycerine (NTG)-induced vasodilation of aorta segments from *ApoE^-/- ^*mice by intratracheal instillation to control solution consisting of 90% saline and 10% BAL fluid (black), nTiO_2 _(green), fTiO_2 _(blue), or pTiO_2 _(red)**. The measurements are performed in A) absence or B) *ex vivo *presence of the SOD mimic, tempol. The response is expressed as the percent vasodilation of the pre-contraction tension produced by PGF_2_α. Each point on the curves represents the cumulative response at each concentration of the vasodilator. Data are expressed as means ± SEM (n = 10-11).* P < 0.05 (ANOVA) compared with E_max _or EC_50 _values in aorta segments of control mice.

The endothelium-independent vasodilation was investigated as the vasodilatory response of aorta segments to the NO-donor nitroglycerin (NTG) or felodipine (FD; blocks the voltage-dependent calcium channels). The vasodilatory response to NTG indicated no difference between the groups of exposed animals, whereas the *ex vivo *tempol treatment was associated with 14% (95% CI: 1.1 - 26%) lower E_max _value and 3.2 (95% CI: 1.8 - 5.6) fold higher EC_50 _value than the segments that were not treated with tempol (table 2, Figure 4).

**Figure 5:**
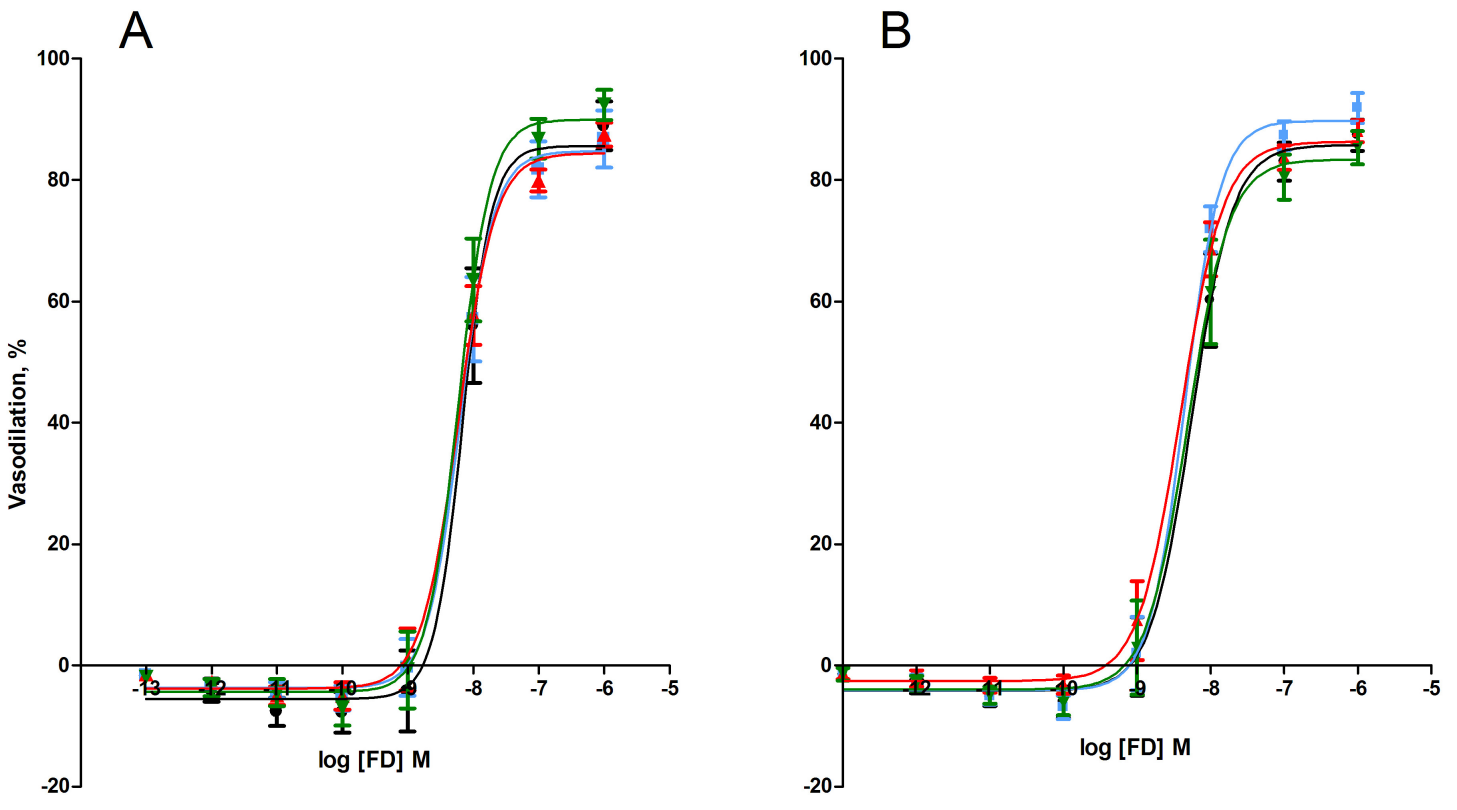
**Felodipine (FD)-induced vasodilation of aorta segments from *ApoE^-/- ^*mice exposed by intratracheal instillation to control solution consisting of 90% saline and 10% BAL fluid (black), nTiO_2 _(green), fTiO_2 _(blue), or pTiO_2 _(red)**. The measurements are performed in A) absence or B) *ex vivo *presence of the SOD mimic, tempol. The response is expressed as the percent vasodilation of the pre-contraction tension produced by PGF_2_α. Each point on the curves represents the cumulative response at each concentration of the vasodilator. Data are expressed as means ± SEM (n = 10-11).

The vasoactive response to the calcium channel antagonist FD was not affected by the *in vivo *exposure to any particle as compared to the control suspension and there was no difference in the vasodilation in the presence or absence of tempol (table 2, Figure 5).

The addition of the nitric oxide synthase inhibitor N'-monomethyl L-arginine (L-NMMA) increased the vasocontraction in the pre-constricted aorta segments, whereas there was no difference in regard to either exposure of animals to particles or *ex vivo *presence of tempol (table 3).

**Table 3:** Excess vasocontraction (in %) induced by N'-monomethyl L-arginine (L-NMMA, 0.10 mM) after PGF_2α _precontraction (1 μM) in aorta segments from *ApoE*^-/- ^mice after intratracheal instillation of TiO_2 _particles or control solution (90% saline and 10% BAL fluid).

Exposure i.t. instillation	Drug	- tempol	+ **tempol**
Control	L-NMMA	17.0 ± 2.84	11.3 ± 1.08
fTiO_2_		10.2 ± 1.49	14.3 ± 2.62
pTiO_2_		14.4 ± 2.89	13.2 ± 2.21
nTiO_2_		12.4 ± 1.65	15.9 ± 3.67

The measurements were done in both absence and *ex vivo *presence of the SOD mimic, tempol. Data are expressed as means ± SEM (n = 10-11).

### Plaque progression

**Figure 6:**
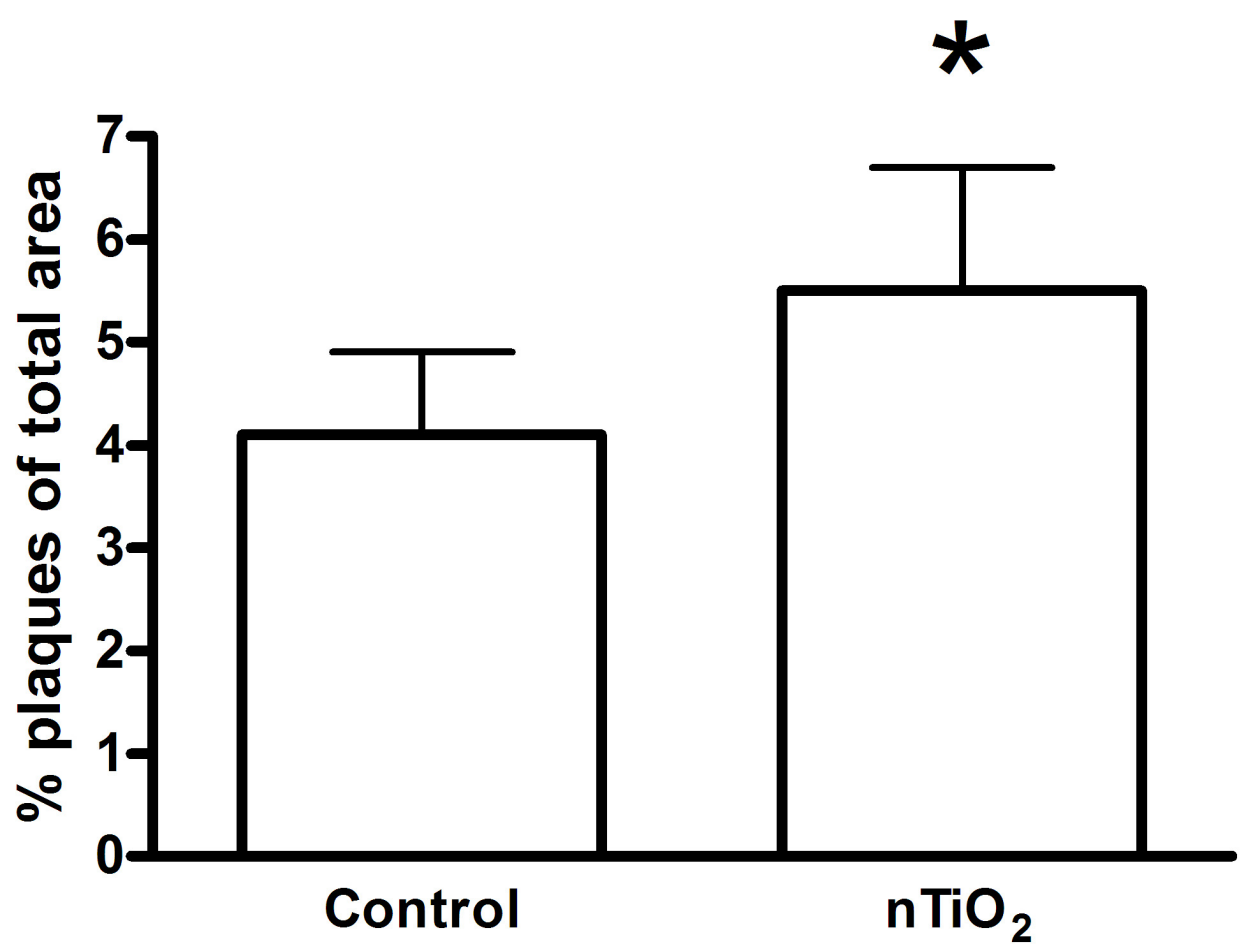
**Plaque area in whole aorta from 20-21 weeks old *ApoE^-/- ^*mice on a regular diet and exposed to control solution (90% saline and 10% BAL fluid) or nTiO_2_**. The plaque areas were determined in the mice after an exposure period of nTiO_2 _(20.6 nm) or control solution once a week by intratracheal instillation for four weeks, followed by a period of five weeks without exposure to particles. The data are expressed as means ± SEM (n = 8). We could not detect plaques in two mice from each group and these are not shown in the figure. * denotes a significant effect on plaque progression in exposed mice vs. the control group.

Figure 6 depicts the data from the assessment of plaque progression in whole aorta tissue of mice exposed to 0.5 mg/kg of nTiO_2 _by i.t. instillation once a week for four weeks giving a total dose of 2 mg/kg, followed by a five week particle-free period before sacrifice. We could not detect any plaques in two mice from each group. The plaque area was statistically significantly higher in the nTiO_2 _exposed mice (5.5 ± 1.2%) compared to the controls exposed to a solution with 90% saline and 10% BAL fluid (4.1 ± 0.8%; P = 0.018, Student's t-test). Inclusion of mice, in which we could not detect plaques in the aorta, in the statistical analysis showed no statistically significant effect (P = 0.096, Mann-Whitney U-test). We regard these results as showing a modestly increased plaque progression in the mice that were exposed to nTiO_2_.

### Levels of mRNA of Mip-2, Mcp-1, Icam-1, Vcam-1 and Vegf in lung tissue

The mRNA levels of *Mip-2*, *Mcp-1*, *Icam-1*, *Vcam-1*, and *Vegf *in the lung tissue of mice exposed twice (26 and 2 hours before sacrifice) to nTiO_2_, pTiO_2_, or fTiO_2 _are reported in table 4. There was no difference in the mRNA levels between the control group and particle exposed mice. The results from the study on repeated exposure to nTiO_2 _(sacrificed five weeks after the last exposure) are shown in table 5. This exposure was also associated with unaltered mRNA levels of *Mip-2*, *Mcp-1*, *Icam-1*, *Vcam-1*, and *Vegf *in the lung tissue.

**Table 4:** Inflammatory markers measured in mice having received two intratracheal instillations of either control bronchoalveolar lavage (BAL) suspension or 0.5 mg/(kg bodyweight) of one of the particle suspensions; fTiO_2_, pTiO_2_, or nTiO_2_, separated by 24 hours (n = 10-11).

Exposure i.t. instillation	mRNA expression level (Median (quartile 25-75%))				
	*Mcp-1 *(*10^-9^)	*Mip-2 *(*10^-9^)	*Icam-1 *(*10^-6^)	*Vcam-1 *(*10^-6^)	*Vegf *(*10^-6^)

Control	54.5 (27.7-89.3)	84.5 (41.5-150.6)	40.8 (27.4-70.2)	0.62 (0.32-0.88)	96.9 (59.3-271.2)
fTiO_2_	24.4 (9.9-38.9)	71.9 (35.2-111.6)	32.8 (29.0-54.4)	0.34 (0.19-0.60)	99.7 (74.2-109.4)
pTiO_2_	37.1 (24.3-52.8)	77.8 (36.2-140.0)	30.3 (25.7-55.2)	0.38 (0.19-1.04)	105.1 (90.3-168.0)
nTiO_2_	27.3 (20.7-64.9)	49.1 (39.0-237.9)	38.9 (18.3-66.8)	0.36 (0.27-0.63)	80.1 (47.0-144.1)

Expression of mRNA is normalised to 18S rRNA.

**Table 5:** Inflammatory markers measured in mice exposed to nTiO_2 _(0.5 mg/kg) or control bronchoalveolar lavage (BAL) suspension by intratracheal instillation once a week for four weeks and sacrificed 5 weeks later (n = 10).

Exposure i.t. instillation	mRNA expression level (Median (quartile 25-75%))				
	*Mcp-1 *(*10^-9^)	*Mip-2 *(*10^-9^)	*Icam-1 *(*10^-6^)	*Vcam-1 *(*10^-6^)	*Vegf *(*10^-6^)

Control	141.9 (98.3-166.5)	43.2 (39.3-53.0)	21.8 (17.4-39.0)	0.30 (0.13-0.50)	0.70 (0.45-1.18)
nTiO_2_	265.9 (80.6-359.5)	53.3 (25.4-140.2)	38.9 (18.6-56.9)	0.40 (0.23-0.83)	0.90 (0.78-1.38)

Expression of mRNA is normalised to 18S rRNA.

### Assessment of NO response in HUVECs

**Figure 7:**
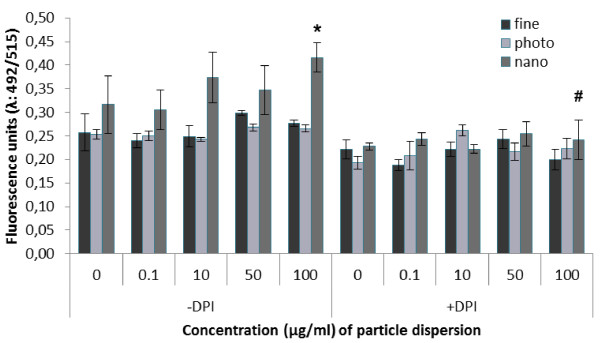
**Nitric oxide (NO) production in human umbilical vein endothelial cells (HUVECs) exposed to three physicochemically different TiO_2 _particles and compared to control cells**. The NO production was measured in absence or presence of the NO synthase inhibitor diphenyleneiodonium chloride (DPI). Values are means ± SEM. * P < 0.05 control versus nTiO_2_. ^# ^P < 0.05 significant treatment effect within group.

The exposure to nTiO_2 _particles was associated with a 32% (95% CI: 23% - 42%) increased level of NO in HUVEC cultures treated with 100 μg/ml (P < 0.05, ANOVA) (Figure 7). The NO levels were unaltered by co-incubation with diphenyleneiodonium chloride (DPI), which is a non-selective inhibitor of NO synthase activity. This abolished the particle-induced NO production in cells incubated with nTiO_2 _(P < 0.001, ANOVA). The incubation with fTiO_2 _or pTiO_2 _did not increase the level of NO (P < 0.01 and P < 0.05 for cultures treated with fTiO_2 _and pTiO_2_, respectively).

**Figure 8:**
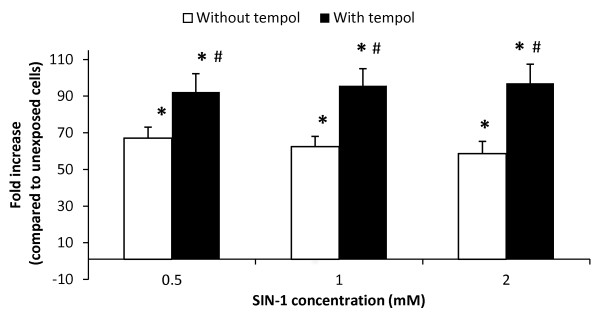
**Nitric oxide (NO) production in human umbilical vein endothelial cells (HUVECs) exposed to the NO donor SIN-1 for a total of three hours in the absence or presence of 1 mM tempol**. Values are means ± SEM. * P < 0.05 SIN-1 versus unexposed cells. ^# ^P < 0.05 for difference between SIN-1 generated NO in the absence or presence of tempol.

We assessed a possible NO scavenging effect of tempol in HUVECs exposed to 3-morpholinosydnonimine (SIN-1) (Figure 8) or NTG. There was a strong single-factor effect of the SIN-1 exposure, showing increased NO production at all concentrations as compared to the unexposed cells (P < 0.001, single-factor effect of SIN-1). The concentrations of SIN-1 were relatively large and there were more than 60-fold larger NO levels compared to the unexposed cells. This probably explains the flat concentration-response relationship. A wider concentrations span of SIN-1 (e.g. ten-fold rather than two-fold dilutions) might have yielded a clearer concentration-response relationship. The SIN-1 treated cultures had higher levels of NO in the presence of tempol (P < 0.01, single-factor effect of tempol). These results indicate that tempol preferentially removes superoxide anion radicals and therefore increases the level of NO in the HUVECs. In a different experiment we treated HUVECs with 0.55 mM NTG in the absence or presence of 1 mM tempol. HUVECs that were treated with NTG, in the absence of tempol, had a 1.45 (95% CI: 1.38-1.51) fold larger NO level as compared to the unexposed control. In the presence of tempol, the NTG treatment increased the NO level by 1.47 (95% CI: 1.34-1.60) fold. NTG was a less potent NO donor than SIN-1, which is clearly observed by the lower production of NO in our experiment, although it was increased relative to the control (P < 0.001, single-factor effect of NTG). The presence of tempol did not affect the NTG-induced NO production in HUVECs (P = 0.16, single-factor effect of tempol). This is possibly because the spontaneously generated superoxide anion radicals in HUVECs are efficiently removed by endogenous SOD.

## Discussion

In this study we show that four repeated i.t. instillations of nTiO_2 _were associated with a modest increase in plaque progression in the aorta of *ApoE*^-/- ^mice, whereas there was little pulmonary inflammation and only minor effects on vasodilatory function of endothelial and smooth muscle cells in segments after two instillations. *Ex vivo *addition of the SOD mimic agent, tempol, reduced the endothelium-dependent vasodilatory function in aorta segments from unexposed mice, whereas tempol increased this function in segments from mice exposed to pTiO_2_. The minimal effect of pulmonary TiO_2 _exposure on vasodilatory function was supported by minimal effects on NO production in HUVECs.

The endothelium and its product NO are key regulators of vascular function. Reduced bioavailability of NO is involved in the initiation and progression of atherosclerosis. The particle exposure of HUVECs showed an increase in the level of NO when the cells were exposed to nTiO_2_. This increase was abolished after addition of DPI, which is a non-selective inhibitor of NO synthase activity in the concentration that we have used. The addition of DPI also blocked the accumulation of NO in HUVECs that were not exposed to particles, indicating that the basal NO production originated from NO-generating enzymes. DPI also inhibits NAD(P)H oxidase, but this probably increases the cellular NO level because of lower possibility of reaction with superoxide anion radicals. iNOS enzymes are expressed during inflammatory conditions, but the one-hour incubation time in our study is probably too short to be associated with upregulation of iNOS. Collectively, the results suggest that the particle-derived NO production originated from eNOS activity.

We investigated the plaque progression in *ApoE^-/- ^*mice exposed to nTiO_2 _once a week for four weeks, followed by a period of five weeks before sacrifice. This particular sample was chosen because previous *in vitro *studies had indicated inflammatory response. Furthermore, Rossi *et al. *showed that a silica-coated TiO_2 _material (similar to nTiO_2_) was the only among several TiO_2 _materials that gave pulmonary inflammation [24]. The protocol used in the present study was similar to the one used for assessment of plaque progression by i.t. instillation of single-walled carbon nanotubes (SWCNT), which had been associated with slightly larger plaque area (9%) in the aorta compared with controls (5.5%) in mice on a high-fat diet [25]. We observed only a modest increase in plaque progression in the aorta of nTiO_2 _exposed mice compared to the control group. This is to the best of our knowledge the first report on plaque progression enhanced by i.t. instillation of nanosized TiO_2 _particles. This observation could be further supported by histology of key sites in the vascular tree or staining of lipids to show fatty streaks/plaques. The exposure was not associated with increased expression of *Mcp-1 *or *Mip-2 *or altered vascular response in terms of *Icam-1*, *Vcam-1 *or *Vegf *mRNA expression levels in the lungs at the time of sacrifice. The mice in our study were fed a regular diet, which might have been associated with slow plaque progression. This is supported by observations that i.t. instillation of carbon black particles was associated with accelerated plaque progression (9.1%) compared to controls (5.5%) in *LDLr^-/- ^*mice on a high-fat diet, whereas the same exposure was statistically non-significantly increased from 2.2% (controls) to 3.0% (exposed) in mice on a regular diet [6]. Similar findings have been observed in *ApoE^-/- ^*mice exposed by inhalation to concentrated ambient air particles where the progression of atherosclerosis was largest in mice on a high-fat diet [26,27]. The plaque progression in mice on a low-fat diet was statistically non-significantly increased in the aorta from 13.2% (filtered air) to19.2% (concentrated ambient air particles) [26] and 10.4% (filtered air) to 14.2% (concentrated ambient air particles) after six months exposure [27]. It is possible that a high-fat diet and a longer exposure period would have rendered the mice more susceptible to particle-induced plaque progression. The mechanisms leading to accelerated plaque progression by inhalation of concentrated ambient air particles involves pro-oxidant and inflammatory milieu in the vasculature, which seems to occur in animals without pulmonary inflammation [28]. It has been shown that accelerated progression of atherosclerosis in *ApoE^-/- ^*mice on a regular diet occurred concomitantly with loss of HDL anti-inflammatory property and systemic inflammation (assessed as elevated hepatic lipid peroxidation) after inhalation of fine or ultrafine concentrated ambient air particles at a concentration that did not generate pulmonary inflammation [29]. Other studies of exposure to concentrated ambient air particles in *ApoE^-/- ^*mice have shown evidence of NAD(P)H oxidase dependent generation of reactive oxygen species, expression of iNOS and nitrotyrosine in aorta tissue [30].

We *a priori *hypothesized that excess generation of superoxide anion radicals would be implicated in dysfunction of vasomotor response. Thus, we measured the vasomotor function with and without the SOD mimic, tempol. The results from our investigation do not indicate any alterations of the vasomotor function related to particle exposure. We obtained full concentration-effect curves for all included vasodilators, indicating that we have reliable measurements of these responses covering a wide range of concentrations. In addition, aorta segments treated with L-NMMA had increased vasocontraction, whereas there was no difference between vessels from control and particle-exposed mice, indicating that spontaneous vasocontraction did not bias the vasodilatory response. The largest effect on vasomotor function was observed in the measurement of the endothelium-dependent vasodilation, where the exposure to TiO_2 _was associated with a statistically non-significant reduction of the maximal acetylcholine response. Mice exposed to the small anatase pTiO_2 _had lower E_max _(46.1% ± 3.9) than the large rutile fTiO_2 _(E_max _51.8% ± 5.0) and small rutile nTiO_2 _(E_max _54.7% ± 5.0), whereas the mice exposed to the control solution had the largest E_max _value (56.7% ± 3.6). This is in accordance with an earlier study showing no effect on acetylcholine-mediated vasodilation in pulmonary arteries of rats exposed to ultrafine and fine TiO_2 _particles (15 and 140 nm, respectively), whereas the same experiment showed a transient endothelial dysfunction in rats exposed to SRM1648 urban dust [31]. Results from another experiment showed unaltered acetylcholine-induced vasodilation in isolated aorta segments exposed to TiO_2_, further supporting the lack of effect in our study [32]. However, these results are in contrast to data obtained from a different experimental model where the vasomotor function was measured in spinotrapezius arteries following intraluminal infusion of the calcium ionophore A23187. It was demonstrated that spinotrapezius muscle arterioles had unaltered responsiveness to NO, whereas the endothelial dysfunction depended on the NO bioavailability [33]. Several studies by the same group have shown that both inhalation and i.t. instillation of fine and ultrafine TiO_2 _were associated with reduced or abolished endothelium-dependent vasodilation in spinotrapezius muscle arteries of normal rats [33-36]. These observations were later extended to coronary arterioles that displayed marked endothelial dysfunction in terms of altered vasodilatory response to shear stress, acetylcholine and the Ca^2+ ^ionophore A23187, whereas the endothelium-independent vasodilatory response to sodium nitroprusside was unaffected [37]. Interestingly, these authors have also reported that the L-NMMA treatment of coronary arterioles led to lower additional increase in vasocontraction in nanosized TiO_2_-exposed rats [38]. The discrepancy between our results and those obtained by the intraluminal infusion of vasodilators might be explained by differences in experimental models, the mode of particle exposure, type of TiO_2 _particles, or species. It has been shown that inhalation of fine or nanosized TiO_2 _was associated with more severe pulmonary inflammation in rats compared to mice and hamsters under conditions where the lung TiO_2 _burdens were equivalent [39,40]. However, we have previously shown that *ApoE^-/- ^*mice had more pulmonary inflammation than wild type mice after i.t. instillation of nanosized carbon black and there was vasomotor dysfunction in the exposed *ApoE^-/- ^*mice as well [41,42].

Most investigations of the effect of tempol have been carried out in animal models of disease, such as hypertension or substantial burdens of atherosclerotic plaques, including studies in old *ApoE^-/- ^*mice showing that treatments with tempol and other SOD-mimicking agents increases the vasodilatory response to acetylcholine [12,43,44]. Tempol has been found to be ineffective as an antihypertensive agent in animal models that are not associated with elevated oxidative stress [45]. We used 12-13 weeks old *ApoE^-/- ^*mice for the vasomotor function experiments, which have the same vasodilatory function as wild type mice if they are not exposed to particles, whereas aged *ApoE^-/- ^*mice are shown to have substantially decreased endothelium-dependent vasodilation in aorta vessels [46,47]. It has been shown that tempol decreased acetylcholine-induced vasodilation in normal rabbits and rats [48,49], whereas it restored acetylcholine-mediated vasodilation in aorta of rabbits pre-treated with an inhibitor of endogenous SOD (diethyldithiocarbamate) and the hypoxanthine/xanthine oxidase superoxide generating system [48]. Similarly, the SOD-mimicking compound (M40430) improved the acetylcholine-mediated vasodilation in *ApoE^-/- ^*mice with compromised vascular function, whereas it was associated with a statistically non-significant 17% decrease in acetylcholine-mediated vasodilation in vessels from wild type mice [50]. The mechanism of endothelial dysfunction in *ApoE^-/- ^*mice involves both decreased bioavailability of NO caused by superoxide anion radicals and degradation of the eNOS cofactor tetrahydrobiopterin [51]. The results from the present study indicate that tempol eliminates vasodilator compounds and/or augments the vascular tone, as have been shown in earlier studies [48,52]. Our results in SIN-1 exposed HUVECs indicate that tempol increased the NO levels by removing superoxide anion radicals, which is in accordance with previously reported observations [53]. This effect of tempol was not observed in NTG exposed HUVECs, which could be because the endogeneous level of SOD efficiently removes the cellular production of superoxide anion radicals. It is possible that exposure to a pure NO donor (e.g. NONOate) and/or inhibition of the endogenous SOD activity would show a clearer effect of tempol. In addition, tempol converts superoxide anion radicals to hydrogen peroxide that has been shown to mediate vasoconstriction in aorta tissue [12,54]. This effect of hydrogen peroxide would be associated with reduced vasodilatory response in our experiment. It seems that the tempol treatment has different effect in healthy and aged/diseased vessels. This possibly limits the extrapolation of our results to effects of tempol-generated hydrogen peroxide in aged/diseased vessels and firm conclusions about the possible role of hydrogen peroxide may require co-incubation with compounds that removes it (e.g. catalase).

Our investigation showed no effect of pulmonary exposure of TiO_2 _particle with different physicochemical characteristics on the vasodilatory function in aorta or mRNA expression of genes relevant in the inflammatory response in the lung, whereas the effect of one form on plaques progression was modest. Therefore, the outcome of the investigation is not optimal for assessment of the importance of particle size or composition or dose. We used mass doses and exposure protocols of TiO_2 _in *ApoE^-/- ^*mice similar to what had shown significant effect for carbon black and SWCNT in terms of vasodilation function and plaque progression, respectively [25,42]. However, a direct dose comparison as mass burden or even surface area, between TiO_2 _particles and SWCNT does not take the overt differences in structure and reactivity into account. The latter exposure was associated with pulmonary inflammatory reaction, systemic oxidative stress and mitochondrial dysfunction, whereas minimal systemic inflammation was detected [25]. TiO_2 _and carbon black are regarded as "low-solubility low-toxicity" particles with similar inflammogenic potency per instilled surface area [55,56]. Indeed, we also found little pulmonary inflammation at doses that were four times larger than the individual doses of fTiO_2 _and nTiO_2_, whereas there was a linear relationship between the surface area and inflammation in relation to fine and nanosized carbon black after a single large-dose exposure [22]. The fTiO_2 _and nTiO_2 _samples in our study also had rutile crystal structure and surface coating, whereas pTiO_2 _was an uncoated mixture of anatase and rutile structure. It has been argued that the anatase crystal structure of TiO_2 _might be more reactive and cytotoxic as compared to rutile crystal structure [57]. In addition, the dispersion protocols might have an influence; some studies on TiO_2 _use dispersions in saline solution [36]. We used BAL fluid, which provides a well dispersed suspension of nanosized TiO_2 _and it has been shown to cause pulmonary inflammation after i.t. instillation in rats, whereas poorly dispersed nanosized TiO_2 _suspensions in saline had similar inflammogenic potency as fine TiO_2 _particles in rats after i.t. instillation [58,59]. However, suspensions in BAL fluid may also lead to formation of a protein corona on the particles, which may change the particle surface chemistry, although we cannot predict whether our TiO_2 _particles would be less potent by coating with proteins.

## Conclusions

We show for the first time that pulmonary exposure to nanosized TiO_2 _is associated with a modest increase in plaque progression. On the other hand, three physicochemically different TiO_2 _nanomaterials (including nanosized TiO_2_) have virtually no effect on vasodilation induced from either endothelial or smooth muscle cells at exposure levels that were not associated with increased *Mcp-1 *and *Mip-2 *expression.

## Materials and methods

### Particles

The following materials were used in this study: fine TiO_2 _(fTiO_2_, RDI-S from Kemira Pigments, Finland) and photocatalytic TiO_2 _(pTiO_2_; W2730X from Kemira Pigments, Finland) both delivered by Beck and Joergensen A/S, Denmark, and nano TiO_2 _(nTiO_2_, UV-Titan L181 from Degussa, Germany) delivered by Boesens Fabrikker ApS, Denmark. A physicochemical characterization has been reported in detail elsewhere [21,22,60].

### Material and Exposure Characterization

The phase composition and crystallite sizes were determined by Monochromated Cu_Kα1 _(1.540598 Å) X-ray diffraction using a Bruker D8 Advance X-ray diffractometer equipped with a Lynxeye CCD detector (Bruker AXS Inc., Madison, WI 53711-5373, USA). Electron microscopy imaging was completed on lacey carbon-coated Cu TEM-grids using a 200 kV Transmission Electron Microscope (TEM) (Tecnai G20, FEI Company, Hillsboro, Oregon, USA) and a Quanta 200 FEG MKII Scanning Electron Microscope [21]. Specific surface area was determined according to DIN ISO 9277 on a Quantachrome Autosorp-1 (Quantachrome GmbH & Co. KG, Odelzhausen, Germany) using multipoint Brunauer, Emmett, and Teller (BET) nitrogen adsorption method after 1 hour degassing at 300°C as a commercial service by Quantachrome GmbH & Co. KG. Elemental composition was analysed by X-ray Fluorescence analysis on a Philips PW-2400 spectrometer as a commercial service by the Department of Earth Sciences, University of Aarhus, Denmark. The organic content was determined indirectly from loss on ignition.

The size distribution of the particle suspensions used for the animal exposures was characterized by dynamic light scattering (DLS) analysis in a Nano Zetasizer (Malvern Instruments, UK). Particle dispersions (0.05 mg/kg bodyweight) were made as described above. Dispersion viscosities were determined using a SV-10 Vibro Viscometer (A&D Company Ltd., Japan). Particle size distributions were measured in disposable polystyrene cuvettes containing 150 μl sample. The optical data were recorded and calculated for both normal and high resolution size distribution using the Dispersion Technology Software v. 5.0 (Malvern Instruments). Particle suspensions were analysed unfiltered and following filtration (3.0 μm filter) and compared with unfiltered BAL solution. Six measurements, consisting of 12-16 scans, were conducted for unfiltered or filtered sample.

### Nitric oxide production in human Umbilical Vein Endothelial Cells (HUVEC)

HUVECs were purchased from Cell Applications (San Diego, CA). The cells were cultured in T75 flasks in Endothelial Cell Growth Medium Kit (Cell Applications, San Diego, CA, USA). Cell cultures were incubated at 37°C in 5% CO_2_-95% air gas mixtures. Media were changed 24-36 hours after seeding and cells were grown to confluence. The cells were used between passages 2-6 because they have morphologic and phenotypic characteristics of endothelial cells.

We used the fluorimetric NOS Detection System DAF-2 DA (Sigma-Aldrich, Schnelldorf, Germany) for the measurement of NO production. The DAF-2 DA probe penetrates cells rapidly where it is hydrolyzed by intracellular esterase to DAF-2 that can react with NO to the fluorescent triazolofluorescein. HUVECs were trypsinated, transferred to a black clear bottom 96-well MT-plate (5*10^5 ^cells/well) and allowed to attach overnight. Particles were added in triplicate in four different concentrations (0.1, 10, 50, and 100 μg/ml) and the cells were incubated at 37°C for 1 hour. The cells were washed briefly and then treated with a reaction mixture, consisting of arginine, DAF-2 DA and Reaction Buffer. The NO production measurement was carried out in the absence or presence of the NOS inhibitor DPI (2 μM). The plates were incubated for 2 hours in the dark before the measurement of triazolofluorescein (excitation filter of 492 nm and an emission filter of 515 nm). All necessary controls, both negative and positive, were included (non-induced, cell-free, without dye, and TNF-α induced with and without DPI).

We investigated whether or not tempol was able to scavenge NO in HUVECs treated with SIN-1 (Sigma-Aldrich, Schnelldorf, Germany) or NTG. The HUVECs were exposed to SIN-1 in absence or presence of tempol (1 mM). We used the same concentration of tempol in this experiment as the concentration in the aorta rings. SIN-1 generates superoxide anion radicals and NO by spontaneous decomposition [53]. It is therefore possible to test the specificity of tempol on NO in our experimental setup. In a different experiment we treated HUVECs with 0.55 mM NTG. The level of NO in HUVECs was determined DAF-2 DA assay.

### Animals

Eleven weeks old female *ApoE*^-/- ^(C57BL/6-Apoe ^tm1^) mice were obtained from Taconic MB (Ejby, Denmark) and acclimatized before entering the experiments. The mice were housed in a temperature- (22-24°C) and moisture- (40-70%) controlled room with a 12:12 h light-dark cycle. All mice were given free access to tap water and standard mouse chow (Standard Altromin no. 1314, Lage, Germany) during acclimatization and housing periods. The experiments were approved by the Danish "Animal Experimental Inspectorate" and carried out following their guidelines for ethical conduct and care when using animals in research.

### Instillation exposure of mice

We assessed the distribution of particles by our i.t. instillation procedure in wild-type mice. This was assessed by i.t. instillation of 1% Evans Blue solution (50 μl/mouse). The lungs were subsequently dried and inflated. In another experiment we instilled 50 μl/mouse of quantum dots (ADSQD621, American Dye Source Inc., Quebec, Canada) and obtained images in a Unit-One dark box using a super sensitive camera (IXON EM+ DU-897 BI, Andor Technologies). The lungs were illuminated through a 600 nm short wave pass filter and the image was obtained through a 620 nm band pass filter. In a third experiment we instilled 40 μl/mouse of radioactive gold particles (18 nm) generated by neutron activation. The images were obtained for 40 min in a single photo emission computed tomography γ-camera (Prism 2000, Phillips) equipped with a pinhole collimator and adjusted for γ-energy of 4000 keV of ^198^Au.

The particles were suspended by sonication in a solution containing 90% sterile, isotonic saline and 10% BAL fluid. The latter was prepared by flushing the lungs of unexposed female *ApoE*^-/- ^mice twice with 0.6 ml isotonic saline. The particle suspensions were sonicated on ice using a Branson Sonifier S-450D (Branson Ultrasonics Corp., Danbury, CT, USA) equipped with a disruptor horn (Model number: 101-147-037). Total sonication time was 16 min, with alternating 10 s pulses ON and 10 s pauses and with amplitude of 10%. Control solutions contained 90% sterile, isotonic saline and 10% BAL fluid from *ApoE*^-/- ^mice. The solutions were divided in aliquots and immediately frozen at -80°C until use. Prior to use, the solutions were thawed at room temperature and vigorously vortexed. We have previously observed that i.t. instillation of saline with 10% BAL fluid was associated with a slightly increased influx of neutrophils, which we have attributed to the instillation procedure rather than the content of BAL fluid [41]. We have not assessed the effect of BAL fluid in regard to vascular endpoints because all the mice in our experiment received vehicle with BAL fluid and the results cannot be biased because of that.

For the assessment of vasodilatory function, four groups of 10-11 mice aged 11-12 weeks received two i.t. instillations of either control solution (90% saline and 10% BAL fluid) or 0.5 mg/(kg bodyweight) of one of the particle suspensions; fTiO_2_, pTiO_2_, or nTiO_2_, separated by 24 hours. We used this dose and exposure period because we have previously observed that exposure to nanosized carbon black was associated with endothelium-dependent vasorelaxation by an identical protocol in *ApoE^-/- ^*mice [42]. The mice were anesthetized using Hypnorm^® ^(fentanyl citrate 0.315 mg/ml and fluanisone 10 mg/ml from Janssen Pharma) and Dormicum^® ^(Midazolam 5 mg/mL from Roche), both mixed with equal volume sterile water. A volume of 0.2 ml was injected subcutaneously in the neck of each mouse before each instillation. The sedated mice were kept on 37°C heating plates prior and subsequent to instillation until recovery from anaesthesia. Two hours after the second dose the mice were sacrificed. To minimize day to day variation, 2-3 particle suspensions were instilled on each exposure day and a control mice was furthermore included each day.

For assessment of effects on plaque progression two groups of 10 mice received 0.5 mg/kg nTiO_2 _or control solution (90% saline and 10% BAL fluid) by i.t. instillation once a week for four weeks and sacrificed 5 weeks later. We used this dose and exposure period because it was similar to a study on SWCNT [25], which we used as a reference condition although we kept the mice on regular chow. We did not use a high-fat diet because we wanted to compare the results from plaque progression experiment with the assessment of vasodilatory function that was carried out on mice fed a regular chow. The plaque progression was not assessed in mice exposed to fTiO_2 _and pTiO_2 _because there was little effect on atherosclerosis in our preliminary experiment with nTiO_2_.

During instillation the mouse was placed supine on an approximately 40 degree slope, where it was held in a favourable position by its front teeth. A light was gently touching the larynx to allow intubation of the trachea with a 24 gauge BD Insyte catheter (Ref: 381212, Becton Dickinson, Denmark) with a shortened needle. The correct placement was verified by a highly sensitive pressure transducer confirming breath and thereby the right placement in the trachea. A volume of 50 μl particle suspension followed by 150 μl air was instilled and the catheter was quickly removed. To ensure that the particle suspension remained in the lungs, the mouse was placed vertically with its head up for 30-60 s.

### Isolation of organs

After the mice were sacrificed the tissues, except the heart and aorta, were immediately removed, quickly frozen in liquid nitrogen, and stored at -80°C until further analysis. The heart and aorta were carefully removed from the animal, stored at ice cold physiological saline solution (PSS: 119 mM NaCl, 25 mM NaHCO_3_, 4.7 mM KCl, 1.18 mM KH_2_PO_4_, 1.17 mM MgSO_4 _7H_2_O, 1.5 mM CaCl_2 _2H_2_O, 0.027 mM ethylene diamine tetraacetic acid and 5.5 mM glucose, pH = 7.4) and dissected free of connective tissue under a light microscope to further use.

### Vasodilatory function

Four aorta segments from each mouse were mounted on steel pins with a diameter 150 μm in the organ chambers of the myograph (multi Myograph 610 M from Danish Myo Technology, Aarhus, Denmark) containing 5 ml cold oxygenated PSS continually perfused with a 95% O_2 _and 5% CO_2 _gas mixture. The distal region of the thoracic aorta was purposely selected to avoid lesions in the segments used for the contractility studies. Each myograph was connected to a computer and the data were collected by the software Myodaq (Danish Myo Technology, Aarhus, Denmark).

The experimental procedure has previously been described in detail [47]. In brief, the temperature in the organ baths were slowly raised to 37°C and the segments were allowed to equilibrate for 30 min. A standard normalization procedure was performed by which the internal lumen diameter of each aortic segment was determined. This procedure enables us to obtain an optimal active tension development in the aortic segments. Preliminary to the specific experiments, it was verified that the aorta segments were variable and that contraction was reproducible. This was done by substituting the PSS in each organ bath with 5 ml warm, oxygenated 125 mM K^+^-PSS (119 mM KCL, 25 mM NaHCO_3_, 4.7 mM KCL, 1.18 mM KH_2_PO_4_, 1.17 mM MgSO_4 _7H_2_O, 1.5 mM CaCl_2 _2H_2_O, 0.027 mM ethylene diamine tetraacetic acid and 5.5 mM glucose, pH = 7.4) followed by 4 times of wash with PSS. This procedure was repeated for a total of three to four times. The K^+^-PSS treatment furthermore ensured that the sympathetic nerve endings were depleted for neurotransmitters. The segments were pre-contracted PGF_2_α (1 μM) before the response to the vasodilators was assessed.

The endothelial function was analyzed by the following vasodilators; acetylcholine (ACh; 10^-10^-10^-5 ^M) and calcitonin-gene related peptide (CGRP; 10^-11^-3*10^-7 ^M). Acetylcholine-induced vasodilation is endothelium dependent as the dilatory effect is exerted via binding to muscarinic receptors (M_3_) on the endothelial cells, whereas CGRP-induced vasodilation is only partly mediated through the endothelium [23]. Furthermore, the endothelium-independent vasomotor response was investigated by using the NO-donor NTG (0^-10^-3*10^-5 ^M), and by using FD (10^-13^-10^-6 ^M), which blocks the voltage-dependent calcium channels on smooth muscle cells.

We tested the effect of particle exposure on vasocontraction in vessels that were treated with L-NMMA (10^-4 ^M), which is a non-selective inhibitor of NOS. In addition, all measurements were done in the absence and presence of tempol (1 mM).

The values of the maximal steady state contraction or dilation (E_max_) and the concentration at which half the E_max _was obtained (EC_50_) were calculated using the GraphPad Prism version 4 (San Diego, CA, USA). Nonlinear regression analysis with equation for sigmoid concentration-response (with variable slope) was used. The results are expressed as the means ± standard error of the mean (S.E.M.).

### Plaque area assessment

**Figure 9:**
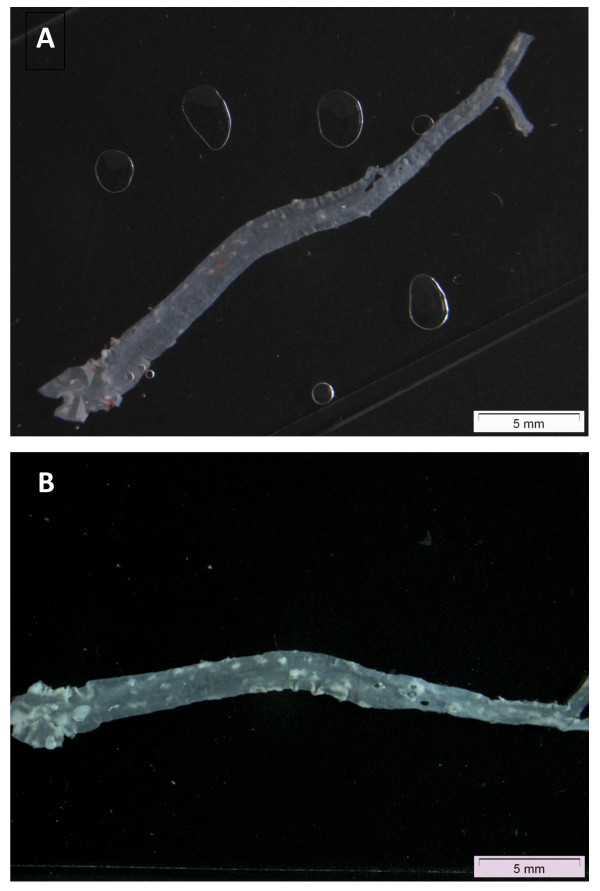
**Representative images of whole aorta tissue from the junction with the heart to the iliac bifurcation from a mouse with undetectable plaque (A) or detectable (B) plaque areas seen as white spots**.

The area of atherosclerotic plaques was determined in the aorta of mice five weeks after four weekly exposures. The entire aorta from the junction with the heart to the iliac bifurcation was placed in a flat bed scanner. The plaques were observed as whitish spots against a black background (Figure 9). The fraction of area of the total aortic surface that contained plaques was determined on a digital microscope image (Digital Imaging Solutions; analySIS^® ^getIT!) by means of the computer software Image J and calculated as % plaques proportional to total aorta area.

### Analysis of mRNA expression levels of Mcp-1, Mip-2, Icam-1, Vcam-1, and Vegf in lungs from ApoE^-/- ^mice

We have previously found close correspondence between *Mcp-1 *and *Mip-2 *mRNA levels in the lung and neutrophil infiltration in BAL fluid in mice exposed to different nanosized particles [41,61,62]. We did not analyse the cell content of cells in the BAL fluid because we did not want to influence the endothelium by the lung infusion and anaesthetic administration that are parts of the BAL fluid collection procedure. We have measured mRNA levels in the lung tissue for *Mcp-1 *and *Mip-2 *as markers of pulmonary inflammation.

*Mcp-1, Mip-2, Vcam-1, Icam-1*, and *Vegf *gene expression was determined using Real-Time Reverse Transcriptase-Polymerase Chain Reaction (RT-PCR). Quantification of the target mRNA was done relative to reference 18S RNA using the relative 2^ΔCt ^method [63]. Total RNA was purified from lung tissue using the TRIzol^® ^Reagent method (Invitrogen A/S, Taastrup, Denmark) and DNase treated as described by the manufacturer (Promega Corporation, Madison, WI) prior to RT-PCR. For cDNA synthesis, less than 1 μg of RNA was used as recommended by Applied Biosystems.

For quantification of the mRNA levels, we used probe and primer solutions (Applied Biosystems,, Foster City, CA, USA) as follows: *Mcp-1 *(AssayID. Mm99999056_m1), *Mip-2 *(AssayID. Mm00436450_m1), *Vcam-1 *(AssayID. Mm01320973_m1), *Icam-1 *(AssayID. Mm00516024_g1), *Vegf *(AssayID. Mm01281449_m1), and 18S rRNA (TaqMan^® ^Ribosomal RNA control reagents: VIC™ Probe). The mRNA levels were quantified in separate wells.

The PCR reactions were performed in triplicate on the Applied Biosystems 7900HT Fast Real-Time PCR System in 10 μl reactions. Four μl of the cDNA preparation was mixed with 98 μl Mastermix (Applied Biosystems, Foster City, CA, USA) and sterile water was added to a final volume of 185 μl. Aliquots of 32 μl were mixed with the respective probe and primer mix solutions. The final concentration of primers and probes were 900 nM and 200 nM, respectively.

### Data analysis

The results are expressed as the means ± standard error of the mean (S.E.M.). Graphs are composed using Graph-Pad Prism by Intuitive Software for Science (San Diego, CA, USA, http://www.graphpad.com). Nonlinear regression analysis with equation for sigmoid concentration-response (with variable slope) was used.

### Statistics

The data on the production of NO by HUVEC were analyzed by three-factor ANOVA test with the concentration, type of particle and *ex vivo *treatment with DPI as categorical variables. The data on vasodilatory function endpoints were analysed by two-factor ANOVA test with the particle and *ex vivo *treatment with tempol as categorical variables. The statistical significance of the tempol treatment is reported as single-factor effects. The mRNA expressions in lung tissue were analyzed by Kruskal-Wallis or Mann-Whitney U-test because of unequal variance between the groups. All tests were accepted as statistically significant at 5% level. The statistical analysis was performed in Statistica version 5.5 (StatSoft Inc., Tulsa, OK, USA).

## Abbreviations

ACh: acetylcholine; apoE: apolipoprotein E; CGRP: calcitonin-gene related peptide; eNOS: endothelial nitric oxide synthase; FD: felodipine; fTiO_2_: fine titanium dioxide; ICAM-1: intracellular adhesion molecule 1; L-NMMA: N'-monomethyl L-arginine; MCP-1: monocyte chemoattractant protein-1; MIP2: macrophage inflammatory protein 2; NO: nitric oxide; nTiO_2_: nano titanium dioxide; NTG: nitroglycerin; pTiO_2_: photocatalytic titanium dioxide; PSS: physiological saline solution; SIN-1: 3-morpholinosydnonimine; SOD: superoxide dismutase; SWCNT: single-walled carbon nanotubes; VCAM-1: vascular cellular adhesion molecule 1; VEGF: vascular endothelial growth factor

## Competing interests

The authors declare that they have no competing interests.

## Authors' contributions

PM, SL, ATS, HW, and UV conceived the study. LM, PM, SL, HW, and UV designed the experiments. LM made the experiments on vasomotor function, assisted and supervised by MS. KAJ made the characterization experiments. NRJ made the experiments on the assessment of particle distribution in mice lungs. LM made the draft of the manuscript, which was revised critically by all authors, who have read and approved the manuscript.
